# Cardiovascular effects of long‐duration space flight

**DOI:** 10.1002/hsr2.2305

**Published:** 2024-08-12

**Authors:** Iqbal Hussain, Rehmat Ullah, Bharti F. N. U. Simran, Parvinder Kaur, Mahendra Kumar, Rohan Raj, Maria Faraz, Amin Mehmoodi, Jahanzeb Malik

**Affiliations:** ^1^ Department of Cardiovascular Medicine Cardiovascular Analytics Group Islamabad Pakistan; ^2^ Department of Medicine Ibn e Seena Hospital Kabul Afghanistan

**Keywords:** astronaut health, cardiovascular effects, long‐duration spaceflight, microgravity adaptation, radiation exposure

## Abstract

**Introduction:**

Early studies exploring the physiological effects of space travel have indicated the body's capacity for reversible adaptation. However, the impact of long‐duration spaceflight, exceeding 6 months, presents more intricate challenges.

**Effects on the Cardiovascular (CV) System:**

Extended exposure to microgravity and radiation profoundly affects the CV system. Notable phenomena include fluid shifts toward the head and modified arterial pressure. These changes disrupt blood pressure regulation and elevate cardiac output. Additionally, the loss of venous compression leads to a reduction in central venous pressure.

**Fluid and Plasma Volume Changes:**

The displacement of fluid from the vascular system to the interstitium, driven by baroreceptor stimulation, results in a 10%–15% decline in plasma volume.

**Cardiac Muscle and Hematocrit Variations:**

Intriguingly, despite potential increases in cardiac workload, cardiac muscle atrophy and perplexing variations in hematocrit levels have been observed. The mechanism underlying atrophy appears to involve a shift in protein synthesis from the endoplasmic reticulum to the mitochondria via mortalin‐mediated mechanisms.

**Arrhythmias and QT Interval Prolongation:**

Instances of arrhythmias have been recurrently documented, although generally nonlethal, in both Russian and American space missions. Long‐duration spaceflight has been associated with the prolongation of the QT interval, particularly in extended missions.

**Radiation Effects:**

Exposure of the heart to the proton and heavy ion radiation pervasive in deep space contributes to coronary artery degeneration, augmented aortic stiffness, and carotid intima thickening through collagen‐mediated processes. Moreover, it accelerates the onset of atherosclerosis and triggers proinflammatory responses.

**Reentry and Postflight Challenges:**

Upon reentry, astronauts frequently experience orthostatic intolerance and altered sympathetic responses, which bear potential hazards in scenarios requiring rapid mobilization or evacuation.

**Conclusion:**

Consequently, careful monitoring of these cardiac risks is imperative for forthcoming missions. While early studies illuminate the adaptability of the body to space travel's challenges, the intricacies of long‐duration missions and their effects on the CV system necessitate continued investigation and vigilance to ensure astronaut health and mission success.

## 1 INTRODUCTION

The groundbreaking space voyages of Yuri Gagarin and John Glenn marked the start of a revolutionary period of exploration for humanity. This exploration encompassed both the internal and external realms of the human body as it ventured into space. However, this excitement was accompanied by a multitude of unforeseen risks and dangers that the human body could face while navigating the confines of low‐earth orbit.^1^, ^2^ These risks persistently captivated scientific investigation up to the present day. As a response, safeguarding the well‐being of astronauts and cosmonauts swiftly became a focal point for both the American and Russian space programs during the early 1960s. Soon after, countries like Europe, Japan, and China also joined this endeavor.

Early research indicated that the human body could indeed adapt to the challenges of spaceflight, although many uncertainties remained regarding potential long‐term consequences.^3^ Notably, one area of concern was the cardiovascular (CV) health of astronauts.^4^ In this context, National Aeronautics and Space Administration's (NASA's) Longitudinal Study of Astronaut Health, a comprehensive study spanning from 1959 to 2010, determined that the experience of shorter spaceflights did not significantly elevate the risk of cardiac issues such as heart attacks, heart failure, strokes, or coronary artery diseases when compared to age, sex, and body mass index‐matched controls.^5^ Nonetheless, the current focus lies not merely in the impact of space presence, but in the implications of extended habitation. NASA's current objectives are centered around sending astronauts to the Moon (by 2024) and subsequently to Mars (projected for the 2030s). The success of these missions hinges upon the capacity of human beings to endure the intense demands of these journeys, the challenges of the return trip, and the process of reacclimatizing to terrestrial conditions upon their return. A critical distinction arises between the effects of short‐term spaceflights lasting less than 6 months—similar to the experiences of Gagarin, Glenn, and International Space Station (ISS) astronauts—and the effects of extended, expedition‐style spaceflights lasting more than 6 months, exemplified by individuals like Scott Kelly.^6^ Understanding and navigating these differences are of paramount importance, particularly in preparation for the potential challenges posed by prolonged space travel, such as the kind that future Mars‐bound explorers are likely to encounter.

## 2 CV PHYSIOLOGY IN SPACE

The space environment, characterized by microgravity (Microgravity is the condition in which people or objects appear to be weightless), radiation exposure, altered circadian rhythms, dietary constraints, sleep disruption, reduced physical activity, and isolation, subjects the human body to an array of physiological and psychological stressors.^1^ These stressors, collectively referred to as the spaceflight milieu, have a profound impact on various bodily systems, particularly the CV system.^7^

The CV adaptations initiated during spaceflight commence immediately upon departure from Earth's gravitational pull, leading to distinctive physiological changes and challenges.^7^ The effects of microgravity become evident as astronauts experience facial puffiness, nasal congestion, and reduced lower extremity fluid volume due to cephalad fluid shifts.^8^ The absence of a gravitational gradient eliminates the need for blood pressure regulatory mechanisms, thus reducing cardiac workload.^9^ Within the initial 24 h of spaceflight, there is a notable decline in central venous pressure (CVP), a parameter indicating cardiac filling pressure.^10^ This reduction is linked to the alleviation of external compression on veins caused by internal viscera and musculature. It's intriguing to observe that CVP decreases concurrently with amplified cardiac chamber volumes and atrial diameter. Despite the seeming contradiction, the simultaneous decrease in CVP aligns with an increase in left ventricular end‐diastolic volume.^11^ This paradox may be clarified by considering changes in transmural CVP, which reflects atrial distension pressure. The profound fluid shift from the lower to the upper body stimulates baroreceptors, inhibiting the renin–angiotensin–aldosterone system and enhancing atrial natriuretic peptide release, leading to a reduction in blood plasma volume by about 10%–15%.^12^

Cardiac remodeling, including atrophy, is observed during extended spaceflights. The concentration of circulating red blood cells (RBCs) increases as plasma volume decreases, prompting the destruction of newly released RBCs as the body seeks homeostasis. Short‐duration spaceflights result in a loss of hematocrit, but long‐duration flights show increased concentrations of both RBCs and hemoglobin, potentially indicating adaptive mechanisms. While short‐duration spaceflight leads to decreased systolic and mean arterial pressure, long‐duration flights paradoxically exhibit an increase in carotid diameter and cardiac output.^13^ This contrast might be attributed to differences in preflight reference postures. Some studies suggest that long‐duration spaceflight could increase cardiac output by up to 35% and 41%, respectively, contradicting earlier findings.^11^ The complex interplay of microgravity‐induced fluid shifts altered CV dynamics, and compensatory mechanisms showcases the remarkable capacity of the CV system to adapt to the space environment. These adaptations are pivotal for astronaut health during missions and offer valuable insights into CV health on Earth. Further research is necessary to comprehensively understand these adaptations and their implications, ultimately contributing to our understanding of human physiology in both terrestrial and extraterrestrial contexts. In microgravity, the lack of gravitational force significantly impacts the distribution of bodily fluids.^11^ On Earth, gravity causes fluids to be pulled toward the lower extremities, but in space, bodily fluids shift toward the upper body and head due to the absence of this force. This phenomenon leads to facial puffiness and increased pressure within the cranial region, potentially affecting vision. Simultaneously, the lower body experiences a decrease in fluid volume, which can result in reduced urine output and dehydration.^12^

The CV system adapts to the microgravity environment by undergoing changes in cardiac structure and function. One prominent change is cardiac atrophy, wherein the heart's muscle mass decreases due to reduced workload. This phenomenon primarily affects the left ventricle, leading to diminished cardiac output and stroke volume.^14^ The heart rate tends to increase to compensate for the reduced stroke volume, which can lead to an elevated resting heart rate during space travel. Table 1 shows CV adaptations in space. The lack of gravitational force prompts alterations in vascular tone and function.^15^ Blood vessels in the lower body constrict, as they no longer need to counteract the effects of gravity to maintain blood pressure against it. This can result in a shift of blood volume from the lower body to the upper body, contributing to the facial puffiness and intracranial pressure observed in astronauts.^16^ Additionally, the endothelial function, responsible for vessel dilation and constriction, can be compromised, potentially impacting blood flow regulation. Fluid balance is perturbed in space due to the redistribution of bodily fluids and the increased fluid losses from sweat and respiration. Astronauts often experience a decrease in total blood volume and a subsequent reduction in plasma volume.^17^ This can lead to orthostatic intolerance upon re‐entry to Earth's gravity, causing dizziness or fainting upon standing.^18^ Maintaining proper fluid balance becomes crucial to prevent dehydration and its associated complications.

**Table 1:** Cardiovascular adaptations during space flight.

Cardiovascular adaptations	Short‐term	Long‐term
Hematocrit	↓ 10%–15%	Unchanged or ↓
Blood volume	↓ 10%–15%	↓ 10%–15%
Cardiac output	↑ 18%–24%	↑ 41%
Stroke volume	↑ 46%	↑ 35%↑
Ventricular size	↓ 10%	↓ 10%
Central venous pressure	↓	↓
Mean arterial pressure	Unchanged	↓ 10%
Heart rate	Unchanged or ↓	Unchanged or ↓
Arterial stiffness	–	↑ 17%–30%

To counteract the adverse effects of microgravity on CV health, astronauts engage in rigorous exercise regimens. Aerobic and resistive exercises are employed to mitigate the loss of CV fitness, muscle mass, and bone density. Astronauts use treadmills to keep fit during their long duration spaceflight missions, much like gym‐goers on the ground do to stay healthy and they workout roughly 2.5 h a day 6 days a week to stay fit in zero gravity. Regular exercise helps maintain cardiac function, preserve bone integrity, and support overall physiological well‐being during extended space missions.^19^

## 3 CARDIOMYOCYTE ALTERATIONS IN SPACE

At the core of cardiac tissue's contractile function lies the cardiomyocyte, a unique muscle cell characterized by its striated appearance, single nucleus, and limited capacity for proliferation.^20^ A cardinal feature of cardiomyocytes is their abundant mitochondria, essential for energy production.^21^ Given their role in cardiac function, any alterations in gene expression within cardiomyocytes due to factors like microgravity or radiation, hold paramount significance, particularly for NASA's pursuits in long‐duration space missions.^22^

Microgravity, a distinctive challenge faced during spaceflight, triggers considerable stress on the heart, demanding cardiac adaptation.^23^ However, the underlying mechanisms driving these adaptations at the cellular level remain largely uncharted. An experiment conducted with rat cardiomyocytes by Feger et al. delves into the cellular changes induced by microgravity.^24^ Their findings suggest that microgravity prompts physiological adaptations by influencing the protein composition and functionality of vital cellular components, including mitochondria, ribosomes, and the endoplasmic reticulum (ER). These adaptations culminate in a decrease in protein synthesis and ultimately lead to atrophy, a phenomenon requiring further exploration.

The way cells react to stress is influenced by various factors such as the specific type of cell, the nature of the stressor, the duration of exposure, and the cell's current proliferation state.^4^ On a proteomic level, three primary stress responses are identified: the cytoplasmic heat shock response, the unfolded protein response (UPR) within the ER, and the UPR within mitochondria.^25^ In the case of cardiomyocytes subjected to simulated microgravity for a duration of 120 h, notable changes were observed. These included an increase in both the quantity and activity of mitochondria, a decrease in components related to ribosomes and the ER, and modifications in the abundance of crucial proteins associated with the folding and synthesis of proteins.^24^ The study highlighted the upregulation of mitochondrial import protein mortalin and AFG3L2, a protein involved in clearing defective components of the electron transport chain. Conversely, the ribosomal components and ER proteins associated with protein synthesis were downregulated.^24^ This observation suggests that cells might shift their focus from rough ER‐mediated protein synthesis to mitochondrial‐based synthesis, possibly facilitated by mortalin, to ensure ATP production under microgravity‐induced stress.^24^ Furthermore, the study noted a decrease in cytoskeletal components that aid in mitochondrial localization, consistent with the pattern of muscle atrophy. Intriguing insights were also drawn from NASA's “Twins Study” conducted in 2019, comparing genetic and physiological differences between a twin on Earth and the other aboard the ISS for 340 days.^6^ Early in the mission, the twin who experienced the spaceflight displayed heightened levels of mitochondrial DNA and RNA in their peripheral blood. This observation might indicate a potential loss of these components, potentially due to factors like atrophy, apoptosis, or the expulsion of mitochondria from lymphocytes. In essence, it suggests that the microgravity environment plays a role in contributing to cardiac muscle atrophy by influencing the protein synthesis pathways within cardiomyocytes.^26^ These intricate cellular responses not only elucidate the mechanisms behind cardiac adaptation in space but also offer vital information for optimizing astronaut health during prolonged missions and advance our understanding of fundamental cellular processes.^27^

## 4 CARDIAC ARRHYTHMIAS DURING SPACE FLIGHT

Long‐term spaceflight poses a significant concern for NASA due to the potential occurrence of cardiac arrhythmias, which involve disruptions in the coordinated electrical impulses responsible for the rhythmic contraction of the heart's atria and ventricles.^28^ Cardiac arrhythmias during spaceflight encompass a spectrum of irregularities, including disorganized electrical activity like atrial or ventricular fibrillation, bradycardia, tachycardia, premature contractions, and other conduction disorders.^29^ The emergence of these arrhythmias compromises the heart's efficient pumping, amplifying the risk of serious consequences such as sudden cardiac death, stroke, CV disease, and even dementia.^30^ While there is limited empirical data on spaceflight‐induced arrhythmias, these disturbances seem to be relatively frequent yet transient.

**Figure 1:**
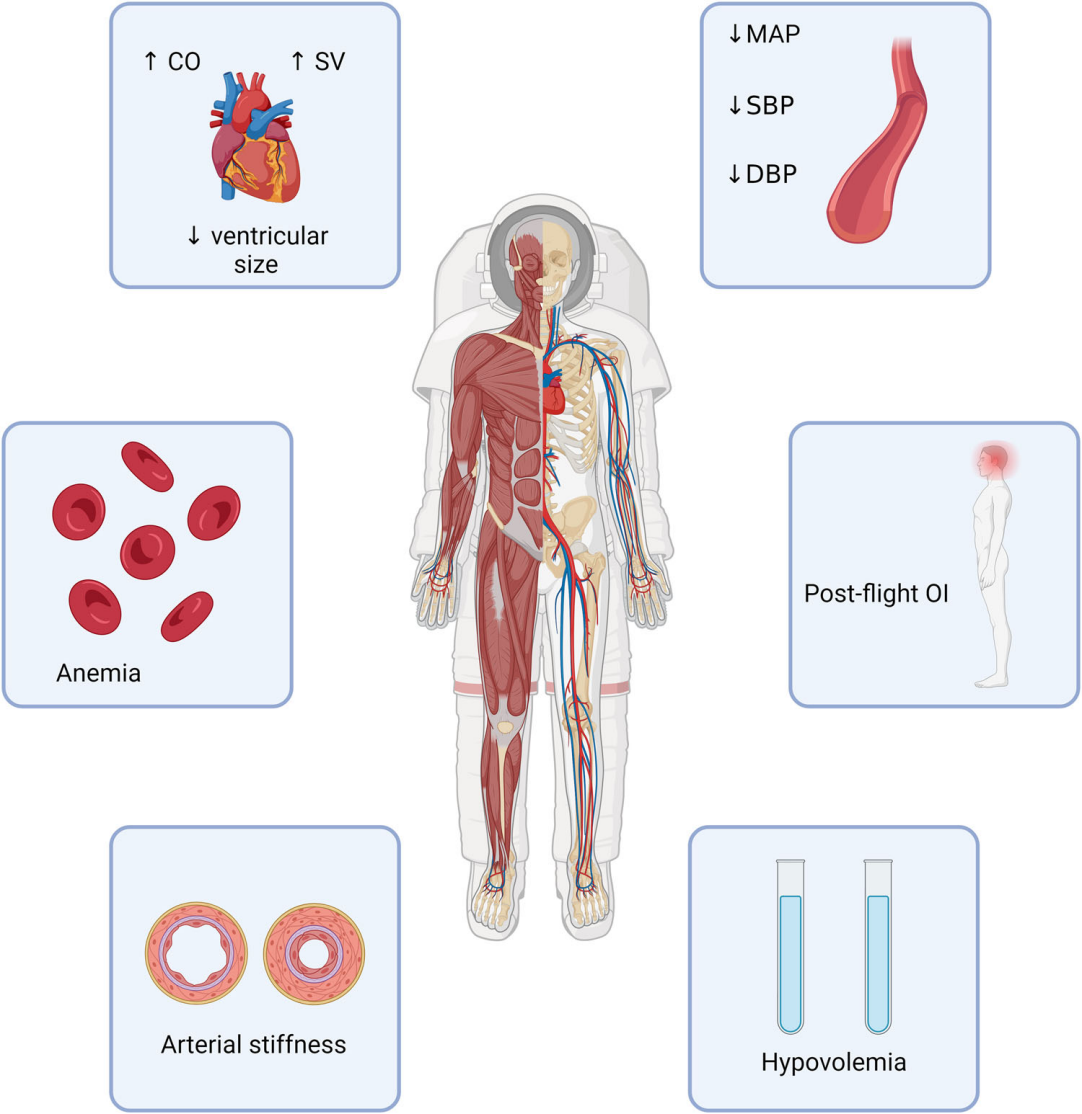
Space flight‐induced cardiovascular effects. CO, cardiac output; DBP, diastolic blood pressure; MAP, mean arterial pressure; OI, orthostatic incompetence; SBP, systolic blood pressure; SV, stroke volume.

The earliest recorded instances of cardiac arrhythmias during space missions date back to the Apollo missions from 1961 to 1972.^31^ Astronaut James Benson Irwin, during the Apollo 15 mission, experienced premature ventricular contractions associated with low potassium levels. Cardiac arrhythmias were sporadically reported during the Skylab missions (1973–1979). The conclusion of the MIR era (1986–2001) saw the Russian Federation reporting a total of 75 arrhythmias and 23 conduction disorders to NASA. One notable case involved a 14‐beat episode of ventricular tachycardia with a peak rate of 215 beats per minute (Figure 1).

While factors like electrolyte imbalances, changes in the autonomic nervous system, and alterations in cardiac chamber mass have been proposed as potential triggers, the exact causative agents remain uncertain.^32^ Moreover, evidence is accumulating regarding conduction issues specific to extended spaceflights. For instance, the extension of the corrected QT interval in electrocardiogram readings, traditionally associated with an elevated risk of Torsades de Pointes—a disorder affecting the repolarization of the heart muscle—and an increased susceptibility to arrhythmias, has been observed in longer spaceflights lasting approximately 4 months.^33^ This effect has not been as pronounced in short‐duration spaceflights. The implications of these findings underscore the significance of investigating the CV impact of prolonged space travel comprehensively. Understanding the occurrence, mechanisms, and potential long‐term consequences of cardiac arrhythmias in microgravity is pivotal for ensuring astronaut health and safety during future missions of extended duration.^34^ It is imperative that the underlying causes of these arrhythmias are elucidated to devise effective preventive and management strategies, safeguarding the CV well‐being of astronauts who undertake the challenging journey of space exploration.^35^, ^36^

## 5 EFFECTS OF COSMIC RADIATIONS

A critical concern for extended space missions is the potential impact of space radiation and cosmic weather on the CV system. Historically, after the atomic bombings in Japan, a substantial portion of ionizing radiation (IR)‐induced mortality was attributed to cardiovascular disease (CVD) and stroke. Similarly, ionizing radiotherapy used in cancer treatment has been associated with inducing CVD.^37^ In healthy individuals and patients, exposure to high doses of IR triggers heart disease, characterized by accelerated atherosclerosis, myocardial fibrosis, valve irregularities, conduction abnormalities, and arrhythmias. These effects can be observed within minutes of radiation exposure, leading to cellular injury. Radiation's specific impact on coronary arteries is also notable.^38^ For instance, exposure to even a single dose of 0.1–0.2 Gy of radioactive iron ions, equivalent to radiation encountered around Earth's orbital plane, has been shown to cause degenerative changes in murine coronary arteries, marked by fibrosis, smooth muscle degradation, and extracellular deposition, observed 15 months postexposure.^39^ Notably, a linear increase in major adverse coronary artery events is associated with administered radiation doses, without a detectable lower or upper threshold.

Considering future Mars missions, a predicted cumulative radiation dose of 0.5–1.0 total Sieverts for an individual traveling to Mars emphasizes the risk.^39^ A 1000‐day Mars mission could increase lifetime death risk due to radiation exposure between 1.3% and 13% for a 40‐year‐old male.^33^ Notably, long‐duration spaceflight and accompanying radiation exposure have been linked to carotid intima‐media thickening. Levels of collagen‐related gene expression and specific collagen chains are increased during spaceflight, suggesting mechanisms underlying the thickening.^40^ The cellular responses to IR are influenced by the type of radiation, and certain cytokines and transcription factors, such as nuclear factor‐κB, can be affected by high‐energy heavy ions (HZE) particles.

**Figure 2:**
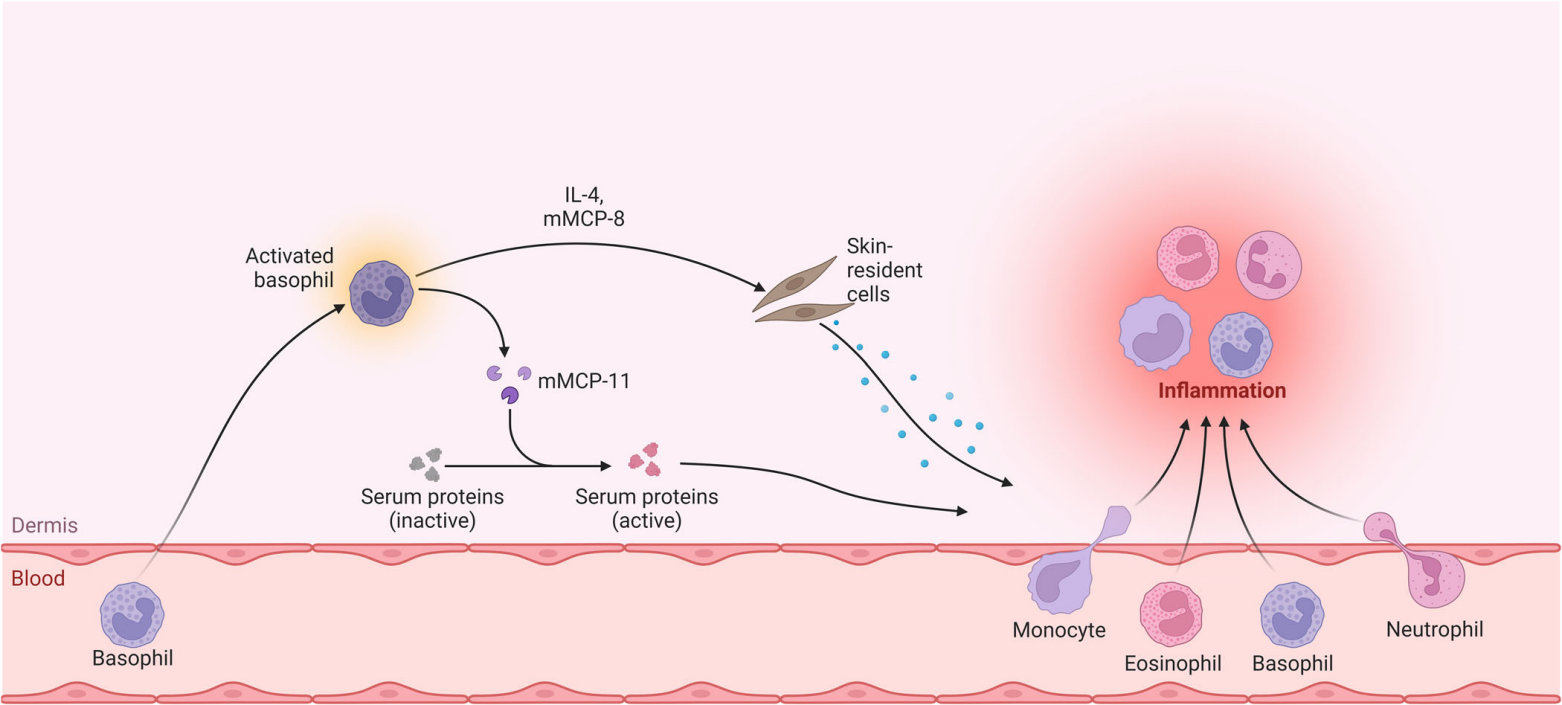
Endothelial cell response to microgravity. IL‐8, interleukin 8; mMCP‐11, mast cell protease 11.

A study involving murine cardiomyocytes investigated the impact of irradiation exposure, revealing changes in pathways related to inflammation, free‐radical scavenging, CV development, and maintenance. Transcription factors like signal transducer and activator of transcription 3, GATA4, and p38 mitogen‐activated protein kinase signaling underwent modulation, indicating overlaps in transcripts associated with neurodegenerative and cardiac muscle disorders. Interestingly, the study noted the upregulation of cardioprotective transcription factors like TBX5, GATA4, and MEF2C even before any signs of cardiac dysfunction became apparent.^41^ However, the manifestation of systolic and diastolic dysfunction occurred later, suggesting a delayed onset of symptoms compared to the modulation of transcription factors. Immune responses to radiation in cardiomyocytes are also implicated. For instance, macrophage infiltration increased twofold 14 days postirradiation, with a slight decrease by Day 28, suggesting a delayed immune response. The specific radiation encountered in space, such as protons and HZE particles, differs from Earth‐based radiation and is anticipated to have more biologically detrimental effects.^42^ The impact of high‐energy protons and radioactive iron ions on cardiac tissue is radiation‐type specific, leading to the migration of immune cells into the heart, DNA oxidation, myocardial fibrosis, and a decline in cardiac function. Furthermore, exposure to particles such as radioactive silicone ions extends the duration of apoptosis and increases the expression of proinflammatory cytokines in the cardiac tissue. It's noteworthy that certain proinflammatory markers identified in the Twins Study were also observed in this context.^6^ Figure 2 shows endothelial cell response to microgravity.

## 6 REACCLIMATIZATION AFTER SPACE TRAVEL

Upon their return to Earth, astronauts commonly experience orthostatic intolerance, marked by symptoms of presyncope such as light‐headedness, dizziness, or nausea. These symptoms are reported in 28%–65% of astronauts during stand or tilt tests after returning, and they could pose challenges even in activities on the surface of Mars, where gravity is only 36% of Earth's.^43^ Orthostatic intolerance might also become a life‐threatening concern during emergency evacuations when re‐exposed to gravitational forces.

There are several reasons for orthostatic intolerance on Earth, such as constriction of restriction arteries, decreases in plasma volume, and stroke volume. Astronauts who are in orbit for extended periods have orthostatic episodes, which can vary from brief tachycardia to orthostatic hypotension, which is defined as a standing systolic blood pressure of less than 70 mmHg, and presyncope.^43^ Interestingly, compared to their counterparts who fly for shorter durations, astronauts who fly for longer durations (129–190 days) exhibit a much higher incidence of orthostatic intolerance.^44^ 20%–30% of astronauts returning from short‐duration flights and 83% of astronauts returning from long‐duration flights, respectively, reported symptoms in earlier studies.^43^, ^44^

Even with NASA's pre‐re‐entry fluid‐loading process, the volume of terrestrial plasma drops by 7%–20% relative to preflight. Static or reverse flow in the internal jugular vein (IJV) is common (55%) by midflight (Day 50) for short‐duration missions, according to a recent study on venous outflow.^45^ Furthermore, it was found that one crew member had an occlusive IJV thrombus and another crew member may have a partial IJV thrombus, indicating problems with blood flow that need to be continuously monitored.^45^ After landing, astronauts from longer spaceflights have larger drops in stroke volume and have more difficulty maintaining upright arterial pressure than astronauts from shorter spaceflights, even though their plasma volume decreases similarly. Long‐duration astronauts also display an attenuated adrenergic response, with five times higher amounts of adrenaline than short‐duration astronauts, and reduced norepinephrine upon attempting to stand. These results point to an acute stress response that was not seen when short‐duration astronauts were readjusting to their environment. During the mission, enhanced imaging and molecular diagnostics may improve astronaut risk assessment.

## 7 CLINICAL IMPLICATIONS

The exploration of space, particularly in the context of extended missions, presents unique challenges to CV health due to the potential impact of space radiation. Understanding the clinical implications of space radiation on the CV system is essential for safeguarding the well‐being of astronauts during and after their missions. Here are some key clinical considerations: 1—CV risk assessment: space agencies and medical teams must conduct thorough CV risk assessments for astronauts before missions. Evaluating individual genetic predisposition, pre‐existing CV conditions, and other risk factors can guide personalized countermeasures and medical interventions. 2— Radiation monitoring and dosimetry: accurate monitoring of radiation exposure during space missions is vital. Implementing dosimetry measures, which quantify the absorbed radiation dose, helps assess the extent of potential CV damage. Real‐time radiation monitoring technologies can provide valuable data for immediate risk assessment. 3— Early detection and screening: astronauts should undergo regular CV screenings before, during, and after space missions. Advanced imaging techniques, biomarker analysis, and electrocardiograms can aid in detecting early signs of radiation‐induced CV changes, enabling timely intervention. 4—Radiation shielding: developing effective radiation shielding strategies for spacecraft is critical. Shielding materials can attenuate radiation exposure and reduce the risk of CV damage. Combining appropriate shielding with spacecraft design considerations can enhance astronaut safety. 5—Pharmacological interventions: exploring pharmacological interventions that mitigate radiation‐induced CV effects is essential. Antioxidant therapies, anti‐inflammatory medications, and drugs targeting specific signaling pathways might help minimize cellular damage and prevent long‐term CV complications. 6—Lifestyle modifications: promoting healthy lifestyle habits among astronauts, both during space missions and afterward, can have a positive impact on CV health. Exercise, a balanced diet, stress management, and adequate sleep contribute to resilience against radiation‐induced damage. 7—Long‐term follow‐up: continued monitoring and follow‐up are crucial components of astronaut health management. Postmission evaluations can track the persistence of radiation‐induced CV changes, informing long‐term medical strategies and potential interventions. 8—Personalized treatment approaches: personalized medicine approaches, including genetic profiling, can guide tailored interventions. Understanding an astronaut's genetic susceptibility to radiation‐induced CV damage allows for targeted countermeasures and treatment plans. 9—Multidisciplinary collaboration: addressing the clinical implications of space radiation requires collaboration between space agencies, medical professionals, radiation physicists, geneticists, and researchers. This multidisciplinary approach ensures a comprehensive understanding of radiation's impact on CV health. 10—Advancements in space medicine: research conducted on the ISS and other platforms provides valuable insights into the effects of space radiation on the CV system. This research informs the development of evidence‐based clinical guidelines and interventions for astronaut health. Figure 1 shows space flight‐induced CV effects.

## 8 FUTURE DIRECTIONS

As the field of space exploration advances, there is a growing imperative to comprehensively address the impact of space radiation on CV health. The challenges posed by long‐duration missions require proactive strategies to mitigate risks and ensure astronaut well‐being. Future research and initiatives will play a pivotal role in advancing our understanding and addressing these concerns. Here are key future directions: 1— Mechanistic studies: continued research is needed to unravel the intricate molecular and cellular mechanisms underlying radiation‐induced CV damage. Investigating how different radiation types interact with CV tissues at various levels, from DNA damage to inflammatory responses, will provide insights into potential therapeutic targets. 2— Biomarker development: the identification of specific biomarkers associated with radiation‐induced CV changes can facilitate early detection and monitoring. Developing a panel of reliable biomarkers that indicate the presence of damage or potential risk will enhance medical surveillance during and after missions. 3—Space‐specific therapeutics: innovative therapeutic approaches tailored to the unique challenges of space radiation are essential. Researchers can explore space‐specific pharmaceutical interventions that counteract radiation‐induced damage while considering the limitations and conditions of space environments. 4—Genetic screening and personalized interventions: advancements in genetics enable personalized interventions based on an individual's genetic susceptibility to radiation‐induced CV damage. Genetic screening can guide customized countermeasures and preventive strategies, minimizing risks for each astronaut. 5—Radiation shielding innovations: efforts to improve radiation shielding technologies will continue. Developing lightweight, efficient, and space‐adapted shielding materials can substantially reduce astronaut exposure to harmful radiation, safeguarding CV health. 6—Premission training and conditioning: astronauts can undergo premission training programs that target CV health. These programs may include exercise regimens, nutrition plans, and stress management techniques to enhance CV resilience against radiation‐induced effects. 7—Long‐term monitoring and data collection: extended postmission monitoring of astronauts is crucial to assessing the long‐term effects of space radiation on CV health. Collecting data over several years will inform the development of accurate risk prediction models and help refine countermeasure strategies. 8. Integration of artificial intelligence (AI) and machine learning (ML): AI and ML technologies can process vast amounts of data from space missions, genetics, and medical assessments. Integrating these technologies can help identify subtle patterns, predict individual responses, and optimize intervention strategies. 9—Collaboration and knowledge exchange: international collaboration among space agencies, researchers, and medical professionals is pivotal. Sharing data, findings, and best practices will accelerate progress in understanding radiation's CV impact and developing effective countermeasures. 10—Holistic health management: future directions should emphasize a holistic approach to astronaut health management. Addressing radiation effects on the CV system should be integrated with strategies for managing other physiological changes, psychological well‐being, and overall astronaut health.

A previously published study provides a detailed and technical account of the physiological effects of long‐term spaceflight, focusing on specific mechanisms such as cephalad fluid translocation, altered arterial pressure, and the role of mortalin in protein synthesis leading to myocyte atrophy.^4^ It emphasizes the intricate CV changes, including reduced plasma volume, decreased CVP, and the occurrence of sublethal arrhythmias, particularly QT interval prolongation. Additionally, it discusses the impact of deep space radiation on coronary artery degeneration, aortic stiffness, carotid intima thickening, and accelerated atherosclerosis.

This review, while covering similar ground, is structured more clearly and concisely, making the information more accessible. It reiterates the effects of microgravity and radiation on fluid shifts, plasma volume, and cardiac muscle atrophy, and highlights the same concerns regarding arrhythmias and QT interval prolongation. It also addresses the long‐term CV risks such as orthostatic intolerance and altered sympathetic responses upon reentry. The primary inference is that the second paragraph presents the same information in a more digestible format, reinforcing and clarifying previously known details while suggesting the need for ongoing vigilance and monitoring of these CV risks in future missions. This streamlined presentation aids in better understanding and highlights the importance of mitigating these risks for the health and safety of astronauts during prolonged space missions.

## 9 CONCLUSION

In the pursuit of understanding the effects of space travel on the CV system, research efforts have spanned from the early days of human spaceflight to the complex long‐duration missions of today. However, despite decades of investigation, our grasp on the full scope of these effects remains incomplete, marked by controversies, contradictions, and gaps in knowledge. This underscores the ongoing necessity for further exploration and inquiry into the intricate interplay between spaceflight conditions and CV health. The challenges of long‐term space travel, characterized by exposure to radiation, altered gravitational conditions, and psychological stressors, necessitate a more comprehensive understanding of their impact on both genetic and epigenetic levels. Unraveling the intricate web of molecular changes induced by these factors is imperative to inform the development of effective countermeasures. By delving deeper into the molecular mechanisms, we can equip organizations like NASA with crucial insights to safeguard astronaut well‐being during voyages to distant planets. Epigenetic alterations induced by space conditions remain a fertile area for investigation. Understanding how radiation, microgravity, and stress influence epigenetic marks can shed light on potential long‐term consequences and help formulate strategies to mitigate them. Likewise, deciphering the genetic underpinnings of space‐induced changes, particularly those related to clonal hematopoiesis and heart function, holds the potential to unveil critical insights for planning extended missions. Collaborative endeavors have extended beyond Earth's confines, with research conducted on the ISS yielding valuable data. Innovations in technology, such as biomolecule‐sensing methods suited for microgravity environments, have the potential to accelerate our ability to capture essential metrics and reduce the interval between measurements, enhancing our grasp of the real‐time impacts on astronaut health. In conclusion, the journey to fully comprehend the effects of spaceflight on the heart remains a continuous odyssey. As we venture into the unknown realms of interplanetary exploration, our commitment to unraveling the complexities of cardiovascular health in space remains unwavering. By addressing controversies, filling gaps in understanding, and collaborating across disciplines, we forge a path toward safeguarding the well‐being of those who dare to traverse the cosmos in the name of human progress.

## AUTHOR CONTRIBUTIONS

**Iqbal Hussain**: Conceptualization; writing—original draft; writing—review and editing; methodology; software; project administration; supervision; investigation. **Rehmat Ullah**: Data curation; resources; project administration; formal analysis; software; visualization; writing—review and editing; writing—original draft; investigation. **Bharti F. N. U. Simran**: Conceptualization; methodology; software; data curation; resources; project administration; visualization; writing—review and editing; writing—original draft. **Parvinder Kaur**: Conceptualization; investigation; writing—original draft; software; formal analysis; project administration; resources. **Mahendra Kumar**: Conceptualization; methodology; software; formal analysis; project administration; resources; supervision; data curation; visualization; writing—review and editing. **Rohan Raj**: Methodology; validation; visualization; writing—review and editing; project administration; formal analysis; software; supervision. **Maria Faraz**: Conceptualization; funding acquisition; writing—original draft; writing—review and editing; visualization; validation; methodology; formal analysis; project administration. **Amin Mehmoodi**: Software; formal analysis; project administration; resources; supervision; data curation; writing—review and editing; visualization; validation; methodology. **Jahanzeb Malik**: Methodology; visualization; writing—review and editing; project administration; software; supervision; data curation; resources; conceptualization; investigation; writing—original draft. All authors have read and approved the final version of the manuscript.

## CONFLICT OF INTEREST STATEMENT

The authors declare no conflict of interest.

## TRASNPARENCY STATEMENT

Amin Mehmoodi (lead author/guarantor) affirms that this manuscript is an honest, accurate, and transparent account of the study being reported; that no important aspects of the study have been omitted; and that any discrepancies from the study as planned (and, if relevant, registered) have been explained.

## Data Availability

Amin Mehmoodi had full access to all of the data in this study and takes complete responsibility for the integrity of the data and the accuracy of the data analysis. No data set available as no new data were generated.

## References

[1] Mann V , Sundaresan A , Mehta S , Crucian B , Doursout M , Devakottai S . Effects of microgravity and other space stressors in immunosuppression and viral reactivation with potential nervous system involvement. Neurol India. 2019;67(suppl):198. 10.4103/0028-3886.259125 31134910

[2] Chaloulakou S , Poulia KA , Karayiannis D . Physiological alterations in relation to space flight: the role of nutrition. Nutrients. 2022;14(22):4896. 10.3390/nu14224896 36432580 PMC9699067

[3] Li YH , Qu LN , Chen HL . Space stress injury and related protective measures. Sheng Li Ke Xue Jin Zhan. 2013;44(5):354‐358. 24475722

[4] Vernice NA , Meydan C , Afshinnekoo E , Mason CE . Long‐term spaceflight and the cardiovascular system. Precis Clin Med. 2020;3(4):284‐291. 10.1093/pcmedi/pbaa022 33391848 PMC7757439

[5] Ade CJ , Broxterman RM , Charvat JM , Barstow TJ . Incidence rate of cardiovascular disease end points in the National Aeronautics and Space Administration Astronaut Corps. J Am Heart Assoc. 2017;6(8):e005564. 10.1161/JAHA.117.005564 28784652 PMC5586420

[6] Garrett‐Bakelman FE , Darshi M , Green SJ , et al. The NASA Twins Study: a multidimensional analysis of a year‐long human spaceflight. Science. 2019;364(6436):eaau8650. 10.1126/science.aau8650 30975860 PMC7580864

[7] Hupfeld KE , McGregor HR , Reuter‐Lorenz PA , Seidler RD . Microgravity effects on the human brain and behavior: dysfunction and adaptive plasticity. Neurosci Biobehav Rev. 2021;122:176‐189. 10.1016/j.neubiorev.2020.11.017 33454290 PMC9650717

[8] Convertino VA . Insight into mechanisms of reduced orthostatic performance after exposure to microgravity: comparison of ground‐based and space flight data. J Gravit Physiol. 1998;5(1):85‐88. 11542376

[9] Shibata S , Wakeham DJ , Thomas JD , et al. Cardiac effects of long‐duration space flight. J Am Coll Cardiol. 2023;82(8):674‐684. 10.1016/j.jacc.2023.05.058 37587578

[10] Masatli Z , Nordine M , Maggioni MA , et al. Gender‐Specific cardiovascular reactions to +Gz interval training on a short arm human centrifuge. Front Physiol. 2018;9:1028. 10.3389/fphys.2018.01028 30108517 PMC6079353

[11] Hargens AR , Richardson S . Cardiovascular adaptations, fluid shifts, and countermeasures related to space flight. Respir Physiol Neurobiol. 2009;169(suppl 1):S30‐S33. 10.1016/j.resp.2009.07.005 19615471

[12] Hughson RL , Helm A , Durante M . Heart in space: effect of the extraterrestrial environment on the cardiovascular system. Nat Rev Cardiol. 2018;15(3):167‐180. 10.1038/nrcardio.2017.157 29053152

[13] Buckey JC , Gaffney FA , Lane LD , et al. Central venous pressure in space. J Appl Physiol. 1996;81(1):19‐25. 10.1152/jappl.1996.81.1.19 8828643

[14] Norsk P . Adaptation of the cardiovascular system to weightlessness: surprises, paradoxes and implications for deep space missions. Acta Physiologica. 2020;228(3):e13434. 10.1111/apha.13434 31872965

[15] Watenpaugh DE . Fluid volume control during short‐term space flight and implications for human performance. J Exp Biol. 2001;204(pt 18):3209‐3215. 10.1242/jeb.204.18.3209 11581336

[16] Watenpaugh DE , Yancy CW , Buckey JC , Lane LD , Hargens AR , Blomqvist CG . Role of atrial natriuretic peptide in systemic responses to acute isotonic volume expansion. J Appl Physiol. 1992;73(4):1218‐1226. 10.1152/jappl.1992.73.4.1218 1447062

[17] Mulvagh SL , Charles JB , Riddle JM , Rehbein TL , Bungo MW . Echocardiographic evaluation of the cardiovascular effects of short‐duration spaceflight. J Clin Pharmacol. 1991;31(10):1024‐1026. 10.1002/j.1552-4604.1991.tb03666.x 1761712

[18] Buckey JC , Lane LD , Levine BD , et al. Orthostatic intolerance after spaceflight. J Appl Physiol. 1996;81(1):7‐18. 10.1152/jappl.1996.81.1.7 8828642

[19] Perhonen MA , Franco F , Lane LD , et al. Cardiac atrophy after bed rest and spaceflight. J Appl Physiol. 2001;91(2):645‐653. 10.1152/jappl.2001.91.2.645 11457776

[20] Später D , Hansson EM , Zangi L , Chien KR . How to make a cardiomyocyte. Development. 2014;141(23):4418‐4431. 10.1242/dev.091538 25406392

[21] Woodcock EA , Matkovich SJ . Cardiomyocytes structure, function and associated pathologies. Int J Biochem Cell Biol. 2005;37(9):1746‐1751. 10.1016/j.biocel.2005.04.011 15950518

[22] Basirun C , Ferlazzo ML , Howell NR , et al. Microgravity × radiation: a space mechanobiology approach toward cardiovascular function and disease. Front Cell Dev Biol. 2021;9:750775. 10.3389/fcell.2021.750775 34778261 PMC8586646

[23] Prasad B , Grimm D , Strauch SM , et al. Influence of microgravity on apoptosis in cells, tissues, and other systems in vivo and in vitro. Int J Mol Sci. 2020;21(24):9373. 10.3390/ijms21249373 33317046 PMC7764784

[24] Feger BJ , Thompson JW , Dubois LG , et al. Microgravity induces proteomics changes involved in endoplasmic reticulum stress and mitochondrial protection. Sci Rep. 2016;6:34091. 10.1038/srep34091 27670941 PMC5037457

[25] Jovaisaite V , Mouchiroud L , Auwerx J . The mitochondrial unfolded protein response, a conserved stress response pathway with implications in health and disease. J Exp Biol. 2014;217(pt 1):137‐143. 10.1242/jeb.090738 24353213 PMC3867496

[26] Fitts RH , Riley DR , Widrick JJ . Physiology of a microgravity environment invited review: microgravity and skeletal muscle. J Appl Physiol. 2000;89(2):823‐839. 10.1152/jappl.2000.89.2.823 10926670

[27] Fitts RH , Riley DR , Widrick JJ . Functional and structural adaptations of skeletal muscle to microgravity. J Exp Biol. 2001;204(pt 18):3201‐3208. 10.1242/jeb.204.18.3201 11581335

[28] Baran R , Marchal S , Garcia Campos S , et al. The cardiovascular system in space: Focus on in vivo and in vitro studies. Biomedicines. 2021;10(1):59. 10.3390/biomedicines10010059 35052739 PMC8773383

[29] Obando MA , Marra EM . Wide QRS Complex Tachycardia. StatPearls Publishing; 2023. 32644480

[30] Harken AH , Honigman B , Van Way 3rd. CW . Cardiac dysrhythmias in the acute setting: recognition and treatment or anyone can treat cardiac dysrhythmias. J Emerg Med. 1987;5(2):129‐34. 10.1016/0736-4679(87)90076-x 3295015

[31] Krittanawong C , Isath A , Kaplin S , et al. Cardiovascular disease in space: a systematic review. Prog Cardiovasc Dis. 2023;81(23):33‐41. 10.1016/j.pcad.2023.07.009 37531984

[32] Atarashi H , Hayakawa H . Etiology and classification of cardiac arrhythmias. Nihon Rinsho. 1996;54(8):2023‐2028 (in Japanese). 8810771

[33] Anzai T , Frey MA , Nogami A . Cardiac arrhythmias during long‐duration spaceflights. J Arrhythm. 2014;30:139‐149. 10.1016/j.joa.2013.07.009

[34] Rossum AC , Wood ML , Bishop SL , Deblock H , Charles JB . Evaluation of cardiac rhythm disturbances during extravehicular activity. Am J Cardiol. 1997;79(8):1153‐1155. 10.1016/s0002-9149(97)00071-4 9114789

[35] Fritsch‐Yelle JM , Leuenberger UA , D'Aunno DS , et al. An episode of ventricular tachycardia during long‐duration spaceflight. Am J Cardiol. 1998;81(11):1391‐1392. 10.1016/s0002-9149(98)00179-9 9631987

[36] Ellestad MH . Ventricular tachycardia during spaceflight. Am J Cardiol. 1999;83(8):1300. 10215307

[37] McDougall JA , Sakata R , Sugiyama H , et al. Timing of menarche and first birth in relation to risk of breast cancer in A‐bomb survivors. Cancer Epidemiol Biomarkers Prev. 2010;19(7):1746‐1754. 10.1158/1055-9965.EPI-10-0246 20570914

[38] Coleman MA , Sasi SP , Onufrak J , et al. Low‐dose radiation affects cardiac physiology: gene networks and molecular signaling in cardiomyocytes. Am J Physiol Heart Circ Physiol. 2015;309(11):H1947‐H1963. 10.1152/ajpheart.00050.2015 26408534 PMC4698384

[39] Boerma M . Space radiation and cardiovascular disease risk. World J Cardiol. 2015;7(12):882‐888. 10.4330/wjc.v7.i12.882 26730293 PMC4691814

[40] Soucy KG , Lim HK , Kim JH , et al. HZE56Fe‐ion irradiation induces endothelial dysfunction in rat aorta: role of xanthine oxidase. Radiat Res. 2011;176(4):474‐485. 10.1667/rr2598.1 21787183

[41] Taunk NK , Haffty BG , Kostis JB , Goyal S . Radiation‐induced heart disease: pathologic abnormalities and putative mechanisms. Front Oncol. 2015;5:39. 10.3389/fonc.2015.00039 25741474 PMC4332338

[42] Ding LH , Shingyoji M , Chen F , Chatterjee A , Kasai KE , Chen DJ . Gene expression changes in normal human skin fibroblasts induced by HZE‐particle radiation. Radiat Res. 2005;164(4 pt 2):523‐526. 10.1667/rr3350.1 16187761

[43] Meck JV , Reyes CJ , Perez SA , Goldberger AL , Ziegler MG . Marked exacerbation of orthostatic intolerance after long‐ vs. short‐duration spaceflight in veteran astronauts. Psychosom Med. 2001;63(6):865‐873. 10.1097/00006842-200111000-00003 11719623

[44] Platts SH , Tuxhorn JA , Ribeiro LC , Stenger MB , Lee SMC , Meck JV . Compression garments as countermeasures to orthostatic intolerance. Aviat Space Environ Med. 2009;80(5):437‐442. 10.3357/asem.2473.2009 19456003

[45] Marshall‐Goebel K , Laurie SS , Alferova IV , et al. Assessment of jugular venous blood flow stasis and thrombosis during spaceflight. JAMA Netw Open. 2019;2(11):e1915011. 10.1001/jamanetworkopen.2019.15011 31722025 PMC6902784

